# Integrated analysis reveals microbiota–metabolite–immune alterations associated with coronary heart disease

**DOI:** 10.3389/fmicb.2026.1726262

**Published:** 2026-04-09

**Authors:** Baoquan Ren, Hong Wang, Yanyan Cao, Zhihua Zhang, Xia Wu, Shushen Ji, Jiangman Zhao

**Affiliations:** 1 Cardiac Care Unit, Qinhuangdao Integrated Traditional Chinese and Western Medicine Hospital, Qinhuangdao, Hebei, China; 2 Hospital Administration Office, Qinhuangdao Integrated Traditional Chinese and Western Medicine Hospital, Qinhuangdao, Hebei, China; 3 Department of Medicine, Shanghai Zhangjiang Medical Innovation Research Institute, Shanghai Biotecan Medical Laboratory Co., Ltd., Shanghai, China

**Keywords:** coronary heart disease, gut microbiota, immune environment, metabolites, phenylacetylglutamine

## Abstract

**Background:**

The relationship between gut microbiota, metabolites, immune environment, and coronary heart disease (CHD) remains incompletely understood.

**Methods:**

This study enrolled 100 non-CHD controls and 302 CHD patients, including 102 with acute coronary syndrome (ACS), 100 with chronic stable angina pectoris (CSAP), and 100 with ischemic cardiomyopathy (ICM). Gut microbiota was analyzed via 16S rRNA gene sequencing, plasma trimethylamine N-oxide (TMAO) and phenylacetylglutamine (PAGln) were measured by mass spectrometry, and flow cytometry was used to assess T, B, and natural killer (NK) lymphocyte subsets.

**Results:**

CHD patients showed reduced gut microbial richness (Chao1 and ACE indices, *p* < 0.05) compared to controls, with enriched *Actinomycetaceae*, *Streptococcaceae*, *Rothia Micrococcaceae*, *Bacilli*, *Dialister* in CHD groups. Predicted microbial functional pathways, including glutathione metabolism, nitrogen metabolism, and porphyrin and chlorophyll metabolism, were significantly reduced in CHD patients based on PICRUSt2 analysis. PAGln levels were significantly higher in CHD especially in ACS patients than in controls (*p* = 0.0016), positively correlating with CHD severity (GRACE score, Spearman *r* = 0.243, *p* < 0.001), while TMAO showed no significant difference. PAGln negatively correlated with total lymphocytes, T cells, and B cells, and was associated with altered abundances of *Parabacteroides*, *Tannerellaceae, Bacilli*, and so on.

**Conclusion:**

CHD is associated with gut microbiota dysbiosis, and reduced microbial richness, which may influence immune homeostasis.

## 1 Introduction

The gut microbiota has emerged as a critical modulator of human health and disease pathogenesis, functioning as a dynamic metabolic organ that profoundly influences host physiology. Accumulating evidence demonstrates that gut microorganisms and their metabolites play indispensable roles in maintaining metabolic homeostasis (Fan and Pedersen, 2021), immune regulation (Yang and Cong, 2021), and nervous system function (Socala et al., 2021). Dysbiosis-pathological alterations in microbial composition and function has been mechanistically linked to diverse conditions including inflammatory bowel disease (Shan et al., 2022), obesity and diabetes (Aron-Wisnewsky et al., 2021; Wu et al., 2020), neurological disorders (Margolis et al., 2021), autoimmune diseases (Zhang et al., 2020), and malignancies (Wong and Yu, 2023).

Cardiovascular disease (CVD), particularly coronary heart disease (CHD) driven by atherosclerotic coronary artery stenosis, remain the leading global cause of mortality. Recent research has established compelling connections between gut microbiota dysbiosis and CHD pathogenesis (Jie et al., 2017; Witkowski et al., 2020; Khuu et al., 2025). Comparative analyses reveal significant compositional differences between CHD patients and healthy individuals, characterized by reduced microbial diversity and altered abundances of specific taxa (Zhu et al., 2018). Gut microbiota-derived metabolites, such as trimethylamine N-oxide (TMAO) (Canyelles et al., 2023; Zhu et al., 2016) and phenylacetylglutamine (PAGln) (Nemet et al., 2020; Zhu et al., 2023) have been shown to influence lipid metabolism, inflammation, and platelet function, all of which are key factors in the development of CHD (Al Samarraie et al., 2023; Wang and Zhao, 2018). Importantly, microbiota-derived metabolites such as trimethylamine N-oxide (TMAO) (Canyelles et al., 2023) and phenylacetylglutamine (PAGln) (Nemet et al., 2020) have been implicated in critical CHD pathways including dyslipidemia, endothelial inflammation, and thrombotic propensity (Al Samarraie et al., 2023; Wang and Zhao, 2018).

Despite these advances, critical knowledge gaps persist. CHD represents a spectrum of clinically distinct entities including acute coronary syndrome (ACS), chronic stable angina pectoris (CSAP), and ischemic cardiomyopathy (ICM)-each with unique pathophysiological features and prognostic implications. Current research predominantly relies on small-sample observational studies that aggregate CHD subtypes, potentially obscuring subtype-specific microbial and metabolic signatures. Moreover, while individual components (microbiota, metabolites, or immunity) have been studied, their dynamic interplay within the “gut-heart axis” remains inadequately explored. Specifically, the tripartite relationship between gut microbial ecology, metabolite production, and systemic immune responses across CHD subtypes is poorly characterized, despite immune dysregulation being a hallmark of atherosclerosis progression.

In this study, we conducted a comprehensive cross-sectional investigation comparing gut microbiota composition, circulating gut microbiota-derived metabolites (TMAO and PAGln), and lymphocyte profiles across rigorously phenotyped CHD subtypes and matched controls. This study aims to delineate subtype-specific perturbations in the gut microbiota-metabolite-immune network, providing novel insights into CHD mechanisms.

## 2 Methods

### 2.1 Study population

**Figure 1:**
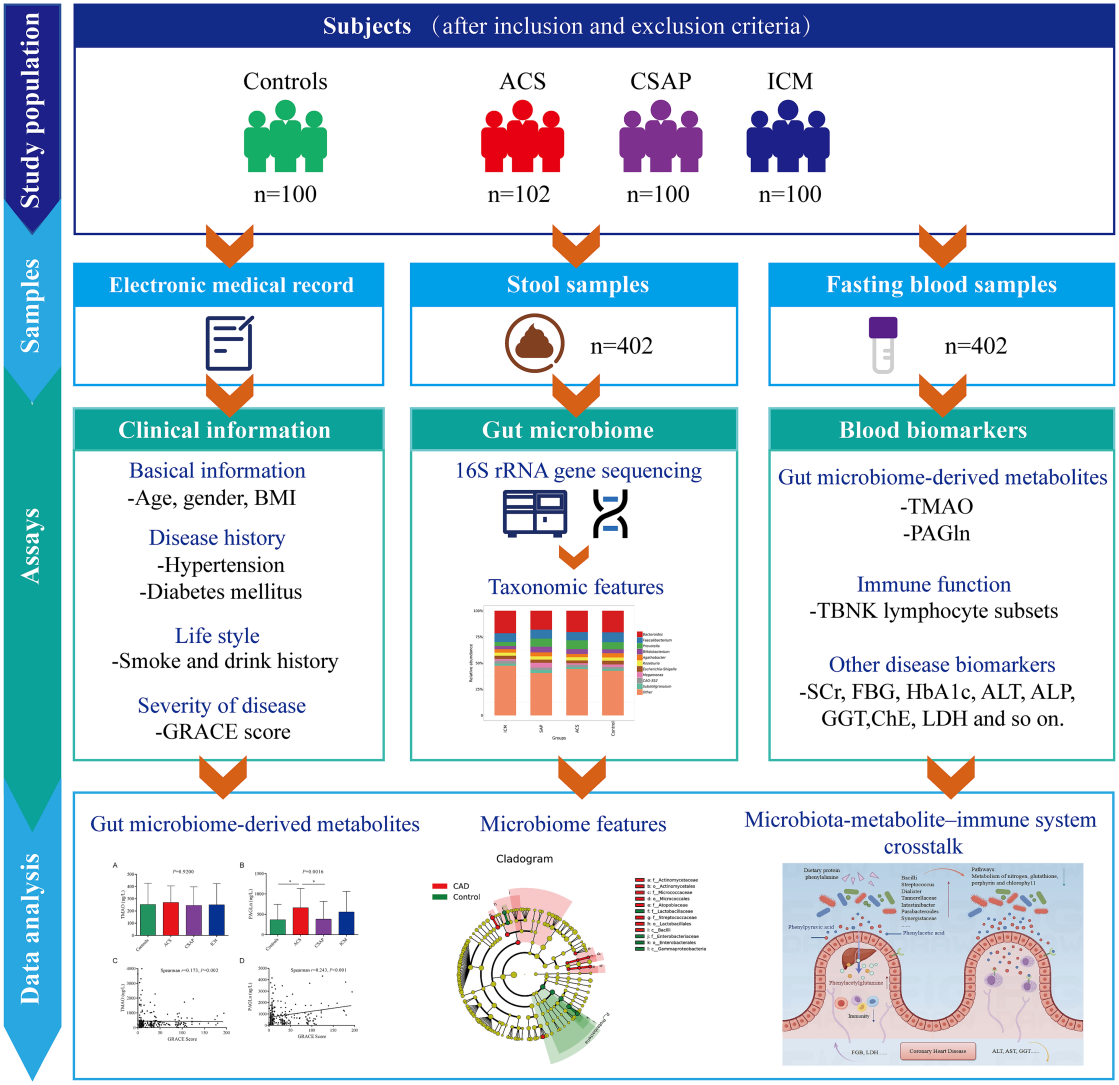
The study work flow and experimental strategy.

This cross-sectional study enrolled a total of 402 participants who visited the Cardiac Care Unit at Qinhuangdao Integrated Traditional Chinese and Western Medicine Hospital, between January and August 2024, including 100 non-CHD controls and 302 CHD patients. The CHD cohort was further stratified into three subgroups: acute coronary syndrome (ACS, *n* = 102), chronic stable angina pectoris (CSAP, *n* = 100), and ischemic cardiomyopathy (ICM, *n* = 100). The inclusion criteria for CHD patients were: (1) diagnosis of CHD by coronary angiography (≥50% stenosis in the main trunk or major branches) and meeting the respective diagnostic criteria for ACS, ICM, or CSAP; (2) age ≥30 years. The non-CHD control group comprised individuals visiting our department or undergoing health examinations due to suspected CHD-related symptoms or cardiovascular risk factors. All control participants were comprehensively evaluated and confirmed to be free of CHD through coronary angiography or coronary computed tomography angiography (CTA). Individuals with obstructive coronary lesions as well as those with non-obstructive atherosclerotic plaques detected on coronary angiography or CTA were excluded from the control group. In addition, all controls were evaluated using electrocardiography and other routine cardiovascular assessments, and individuals with severe cardio-cerebrovascular diseases (e.g., stroke) were excluded. Exclusion criteria for all participants were: (1) other major conditions significantly interfering with test results, as assessed by clinicians (e.g., malignant tumors, hepatic or renal insufficiency, severe infections); (2) pregnancy or lactation; or (3) inability to understand the study purpose or poor compliance. Figure 1 showed the study design. This study received approval from the Ethics Committee of Qinhuangdao Integrated Traditional Chinese and Western Medicine Hospital (Approval No.: KY202403020041), and written informed consent was obtained from all participants.

### 2.2 Clinical and samples collection

Demographic and clinical data were systematically collected from electronic medical records, including baseline characteristics (age, gender, BMI), disease history (hypertension, diabetes mellitus), lifestyle factors (smoking and alcohol consumption), and disease severity metrics. Stool samples in the morning were obtained from all participants for gut microbiome analysis by 16S rRNA gene sequencing. Fasting blood samples were obtained from all participants to assess lipid profiles (total cholesterol, triglycerides, HDL-C, LDL-C), immune cell subsets (T, B, and NK lymphocytes) via flow cytometry, and plasma metabolites (e.g., TMAO and PAGln) through liquid chromatography-mass spectrometry (LC–MS).

### 2.3 Measurement of plasma TMAO and PAGln levels

Venous blood (4 mL) was collected into EDTA-coated tubes from all participants and centrifuged to isolate plasma, which was stored at −80 °C until analysis. Plasma concentrations of TMAO and PAGln were quantified using liquid chromatography–tandem mass spectrometry (LC–MS/MS; AB SCIEX Triple Quad™ 4500MD) at Shanghai Biotecan Medical Laboratory. Briefly, 50 μL of plasma was mixed with 200 μL acetonitrile, followed by vortex mixing, and sequential centrifugation (2,810 × g for 10 min). The supernatant (120 μL) was injected into an Agilent ZORBAX RRHT Eclipse Plus C18 column with a mobile phase of 0.1% formic acid in water (A) and 0.1% formic acid in methanol (B). Analytes were detected in positive-ion multiple reaction monitoring (MRM) mode, and data were processed using MultiQuant 3.0.2 with external calibration curves for quantification.

### 2.4 Lymphocyte subset analysis

Lymphocyte subset analysis was performed using a DxFLEX flow cytometer (Beckman Coulter) and CD3-FITC/CD16 + 56-PE/CD45-PerCP-Cy5.5/CD4-PC7/CD19-APC/CD8-APC-Cy7 fluorescent monoclonal antibody kit (flow cytometry, Beijing Tongsheng Shidai Biotechnology Co., Ltd.). A total of 50 μL of EDTA-K2-anticoagulated whole blood was processed by adding 5 μL of a fluorescent antibody cocktail containing CD3-FITC, CD16 + 56-PE, CD45-PerCP-Cy5.5, CD4-PC7, CD19-APC, and CD8-APC-Cy7. The mixture was incubated in the dark for 20 ± 5 min. Subsequently, 450 μL of 1 × hemolysin was added to lyse erythrocytes, followed by a 15-min incubation. An equal volume of absolute count beads was then added to the sample. Samples were immediately analyzed on the DxFLEX flow cytometer using CytExpert software. Data acquisition continued until either 5,000 events within the lymphocyte gate were recorded or 600 s had elapsed. The percentages of T cells (CD3+), helper T cells (CD3 + CD4+), cytotoxic T cells (CD3 + CD8+), B cells (CD3 − CD19+), and natural killer (NK) cells (CD3 − CD16/CD56+) were determined by gating strategies. Resulting data were exported as CSV files for the calculation of absolute cell counts.

### 2.5 DNA extraction and 16S rRNA gene amplification and sequencing

Total genomic DNA was isolated from fecal samples using the GHFDE100 DNA extraction kit (GUIHE Laboratories, Hangzhou, China), following the manufacturer’s protocol. DNA purity and concentration were verified via NanoDrop ND-1000 spectrophotometry (Thermo Fisher Scientific, USA) and agarose gel electrophoresis. The hypervariable V3–V4 regions of bacterial 16S rRNA genes were amplified using primers 341F (5’-CCTACGGGNGGCWGCAG-3′) and 805R (5’-GACTACHVGGGTATCTAATCC-3′). Amplicons were purified using Agencourt AMPure XP Beads (Beckman Coulter, USA), quantified via PicoGreen dsDNA Assay (Invitrogen, USA), and pooled for paired-end 2 × 250 bp sequencing on the Illumina NovaSeq6000 platform.

### 2.6 Bioinformatics analysis

Raw sequencing data were demultiplexed, merged (Vsearch v2.22.1), and denoised using the UNOISE2 algorithm. Chimeric sequences were removed via *de novo* detection (UCHIME3). Amplicon Sequence Variants (ASVs) were clustered at 100% similarity and taxonomically classified against the SILVA 138 database using QIIME2 (2022.2 release). Low-abundance ASVs (<0.001% of total sequences) were filtered out. Alpha diversity (Chao1, Shannon, Simpson) and beta diversity (weighted/unweighted UniFrac, Bray-Curtis) were calculated in QIIME2. Principal coordinates analysis (PCoA) and permutational multivariate ANOVA (PERMANOVA) were performed to assess group differences. Differential taxa abundance was evaluated using Kruskal-Wallis tests and LEfSe. Functional pathways were predicted via PICRUSt2 and FAPROTAX. All analyses used a significance threshold of *p* < 0.05. No additional adjustment for the covariates such as diabetes, hypertension, smoking, and alcohol consumption was performed in the microbiota analyses, due to these variables well-matched between the CHD and control groups.

### 2.7 Statistics

Statistical analyses and graphing were performed using IBM SPSS Statistics, GraphPad Prism 6, and the R program. Categorical variables are presented as counts and percentages [n (%)], with associations between variables evaluated using the chi-square test or Fisher’s exact test. Missing data were addressed using a complete-case analysis approach. For variables with incomplete records (e.g., hypertension, diabetes mellitus, smoking and drinking status), analyses were conducted based on available data, and the corresponding sample size for each variable is indicated. Continuous variables are expressed as mean ± standard deviation (mean ± SD). For continuous variables conforming to a normal distribution, intergroup comparisons were conducted using independent samples t-tests, while the Mann–Whitney U test and Kruskal-Wallis H test were employed for comparisons between two and more groups when normality assumptions were violated. The relationship between normally distributed continuous variables was assessed using Pearson correlation analysis, whereas Spearman’s rank correlation coefficient was utilized for non-normally distributed variables. Multivariable linear regression analysis was performed with the GRACE score as the dependent variable, PAGln as the independent variable, and adjustment for multiple covariates.

## 3 Results

### 3.1 Clinical characteristic of study population

The clinical characteristics of the study participants are summarized in Table 1. A total of 402 subjects were enrolled, comprising 100 controls and 302 coronary heart disease (CHD) patients. No significant differences were observed between the control and CHD groups in terms of gender distribution (*p* = 0.593), BMI (25.22 ± 3.65 vs. 25.27 ± 3.75 kg/m^2^; *p* = 0.928), hypertension prevalence (58.9% vs. 66.8%; *p* = 0.169), diabetes mellitus (30.0% vs. 33.2%; *p* = 0.768), smoking (11.6% vs. 9.2%; *p* = 0.494), or alcohol consumption (7.3% vs. 6.5%; *p* = 0.783). CHD patients were significantly older than controls (65.42 ± 12.53 vs. 61.72 ± 11.80 years; *p* = 0.01).

**Table 1:** Clinical characteristics of subjects.

Clinical characteristics	Total	Controls (*n* = 100)	CHD (*n* = 302)	*p*-value
Gender	402	100	302	0.593
Male	232	60 (60.0%)	172 (57.0%)	
Female	170	40 (40.0%)	130 (43.0%)	
Age (years, mean ± SD)	64.50 ± 12.44	61.72 ± 11.80	65.42 ± 12.53	0.010
BMI (kg/m^2^, mean ± SD)	25.26 ± 3.72	25.22 ± 3.65	25.27 ± 3.75	0.928
Hypertension	370	95	271	0.169
No	130	39 (41.1%)	90 (33.2%)	
Yes	240	56 (58.9%)	181 (66.8%)	
Diabetes mellitus	385	95	286	0.768
No	258	65 (68.4%)	191 (66.8%)	
Yes	127	30 (31.6%)	95 (33.2%)	
Smoke	393	95	294	0.494
No	354	84 (88.4)	267 (90.8%)	
Yes	39	11 (11.6%)	27 (9.2%)	
Drink	393	96	293	0.783
No	367	89 (92.7%)	274 (93.5%)	
Yes	26	7 (7.3%)	19 (6.5%)	
FBG (mmol/L)	7.15 ± 3.05	6.90 ± 2.96	7.24 ± 3.08	0.031
HbA1c (%)	6.22 ± 1.26	6.05 ± 1.00	6.29 ± 1.34	0.196
Hcy (umol/L)	13.24 ± 8.31	13.13 ± 7.44	13.28 ± 8.60	0.586
SCr (umol/L)	75.31 ± 32.38	74.37 ± 21.04	75.62 ± 35.34	0.507
LDH (U/L)	196.85 ± 201.40	132.10 ± 63.75	220.76 ± 228.01	<0.001
CK (U/L)	101.21 ± 180.02	68.12 ± 116.76	113.43 ± 197.18	<0.001
ALT (U/L)	33.25 ± 30.82	51.57 ± 36.95	27.12 ± 25.79	<0.001
ALP (U/L)	70.33 ± 45.94	61.12 ± 76.82	73.43 ± 28.49	<0.001
AST (U/Lalt)	30.68 ± 64.27	37.77 ± 92.26	28.31 ± 51.64	0.110
GGT (U/L)	1562.86 ± 5835.21	3982.84 ± 4373.30	753.45 ± 6041.61	<0.001
ChE (U/L)	7131.21 ± 3571.23	4741.60 ± 4653.52	7927.74 ± 2702.65	<0.001
TP (g/L)	63.35 ± 11.70	55.45 ± 13.72	65.97 ± 9.63	<0.001
ALB (g/L)	39.54 ± 7.65	34.77 ± 9.88	41.12 ± 5.99	<0.001
ADA (U/L)	10.82 ± 5.23	8.55 ± 3.55	11.58 ± 5.48	<0.001

Patients were not presented who missed hypertension, diabetes mellitus, smoking and drinking status record.

### 3.2 Comparison of gut microbiota composition among CHD patients and controls

**Figure 2:**
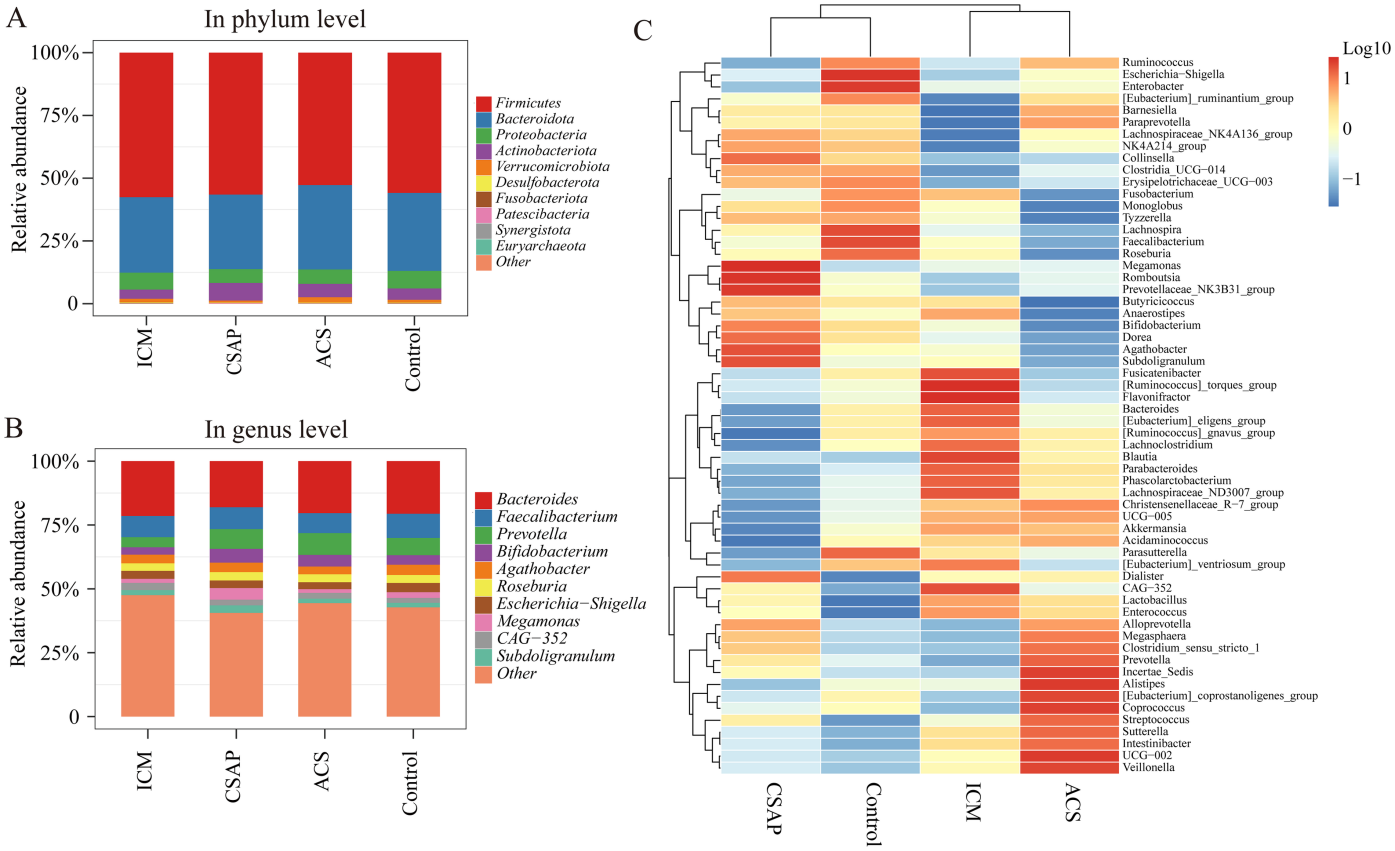
Gut microbiota composition across coronary heart disease (CHD) subtypes and controls. Relative abundance of gut microbial in phylum **(A)** and genus **(B)** level in ischemic cardiomyopathy (ICM), chronic stable angina pectoris (CSAP), acute coronary syndrome (ACS), and control groups. **(C)** Heatmap of microbial genus-level abundance across groups. Hierarchical clustering reveals clustering patterns of genera that distinguish CHD subtypes from controls.

Figure 2 showed the composition of microbiota among CHD patients and controls at the phylum and genus level. At the phylum level (Figure 2A), all groups were dominated by *Firmicutes* and *Bacteroidota*, with minor contributions from *Proteobacteria*, *Verrucomicrobiota*, and others, showing overall structural similarity but subtle variations in less abundant phyla. At the genus level (Figure 2B), *Bacteroides*, and *Faecalibacterium* were prominent. The heatmap (Figure 2C) revealed differential genus abundances, with ACS and ICM clustering distinctly from CSAP and Controls.

### 3.3 Differences in diversity among CHD patients and controls

**Figure 3:**
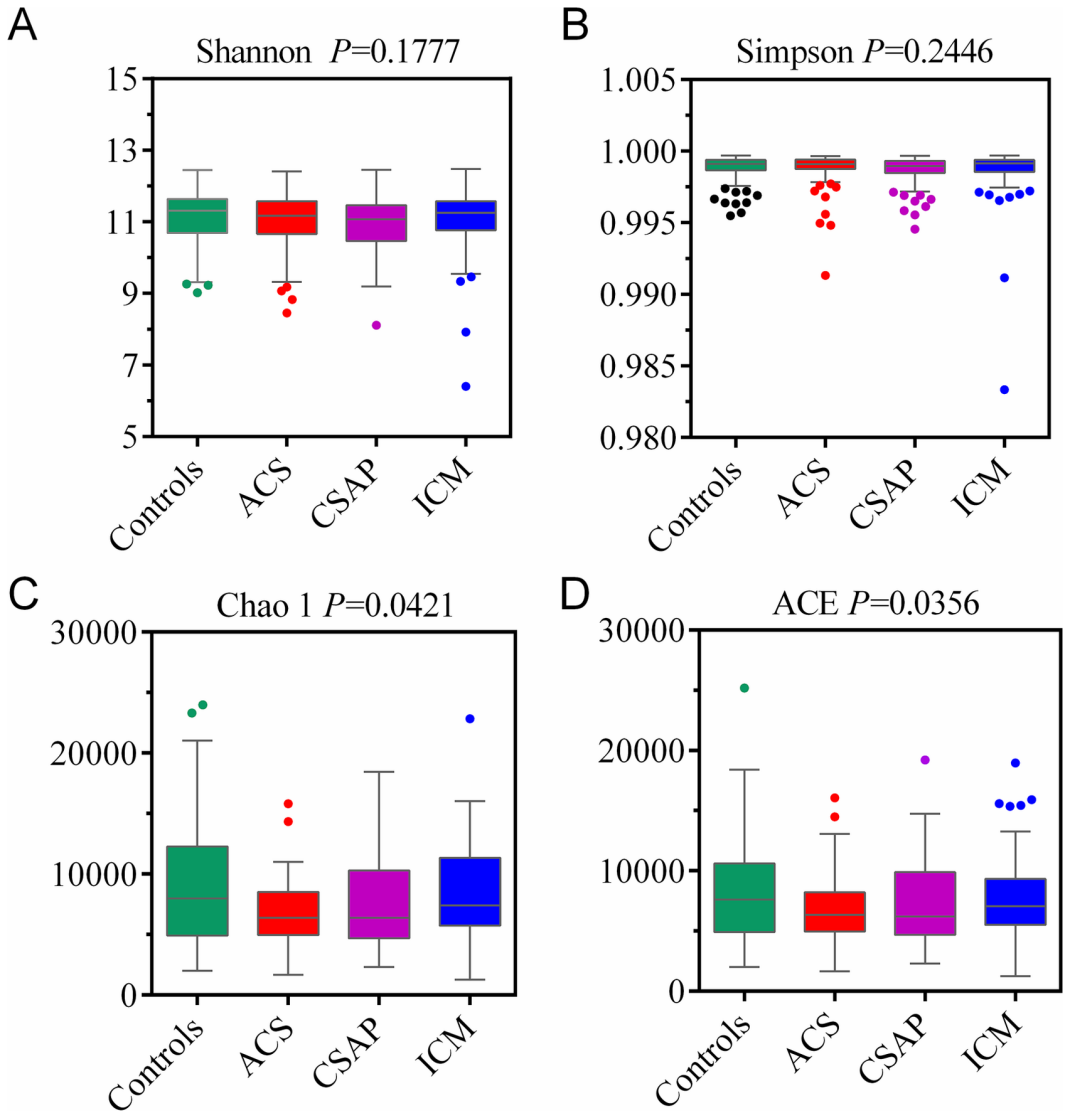
Comparison of Alpha diversity of gut microbiota among coronary heart disease (CHD) subtypes and controls using Kruskal–Wallis *H-*test. **(A)** Shannon index showing microbial community diversity. **(B)** Simpson index reflecting microbial community evenness. **(C)** Chao 1 index indicating microbial richness. **(D)** ACE index also representing microbial richness.

Alpha diversity indices were compared across groups (Figure 3). For Shannon diversity (Figure 3A, *p* = 0.1777) and Simpson diversity (Figure 3B, *p* = 0.2446), no significant differences were observed in microbial community diversity, respectively, among the groups. However, Chao1 (Figure 3C, *p* = 0.0421) and ACE (Figure 3D, *p* = 0.0356) indices, which estimate species richness, revealed significantly higher microbial richness in the Control group compared to ACS, CSAP, and ICM (*p* < 0.05). Boxplots showed that Controls had greater richness (higher medians for Chao1 and ACE), with ACS samples exhibiting the lowest richness. These results indicate that while microbial diversity is comparable across groups, non-CHD controls harbor a more species-rich gut microbiota than CHD subgroups, suggesting potential richness loss in CHD patients.

### 3.4 Taxonomic differences between CAD and control groups

**Figure 4:**
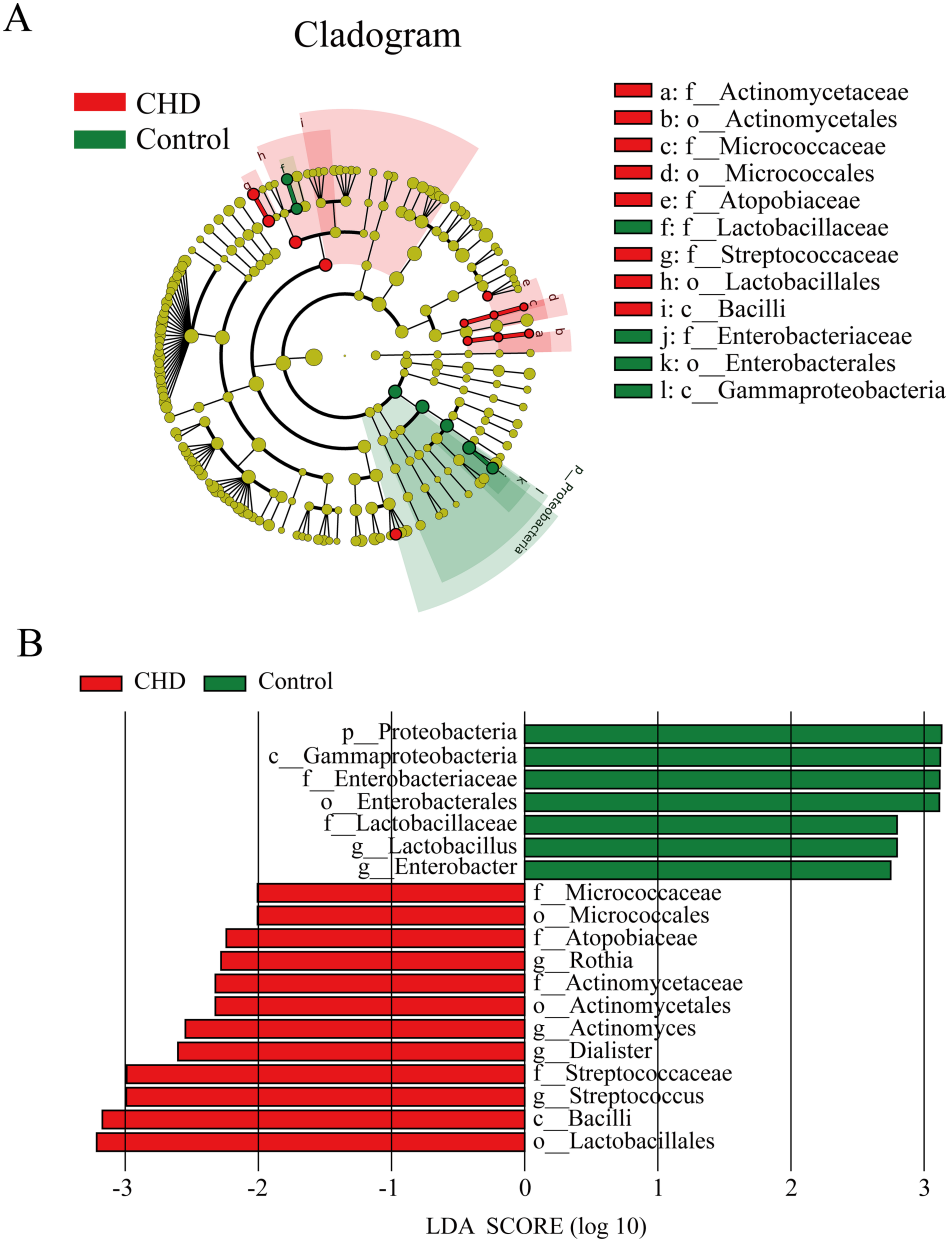
Taxonomic differences in gut microbiota between coronary heart disease (CHD) patients and controls. **(A)** Cladogram representing taxonomic levels (from phylum to genus) with significant differences. **(B)** Linear discriminant analysis (LDA) effect size (LEfSe) plot displays taxa with LDA scores > 2 that are significantly discriminative between CHD and control groups.

To identify taxonomic biomarkers distinguishing coronary artery disease (CAD) patients from controls, we performed linear discriminant analysis effect size (LEfSe) analysis. The cladogram (Figure 4A) visualized the hierarchical taxonomic distribution (from phylum to genus) of differentially abundant taxa. In the CAD group, enriched taxa included *Actinomycetaceae*, *Actinomycetales*, *Micrococcaceae*, *Atopobiaceae*, *Streptococcaceae*, *Lactobacillales*, and *Bacilli*. In contrast, the control group showed enrichment of *Lactobacillaceae*, *Enterobacteriaceae*, *Enterobacterales*, and *Gammaproteobacteria*. The LDA score plot (Figure 4B) quantified the effect size of these taxonomic differences. Taxa enriched in controls included *Proteobacteria* (phylum), *Gammaproteobacteria* (class), *Enterobacteriaceae* (family), *Enterobacterales* (order), *Lactobacillaceae* (family), and *Lactobacillus* (genus), with LDA scores >2, indicating strong discriminative power. For the CAD group, key enriched taxa were *Micrococcaceae* (family), *Micrococcales* (order), *Atopobiaceae* (family), *Rothia* (genus), *Actinomycetaceae* (family), *Actinomycetales* (order), *Actinomyces* (genus), *Streptococcaceae* (family), *Streptococcus* (genus), *Bacilli* (class), and *Lactobacillales* (order), also with LDA scores >2.

### 3.5 Microbial co-occurrence network

**Figure 5:**
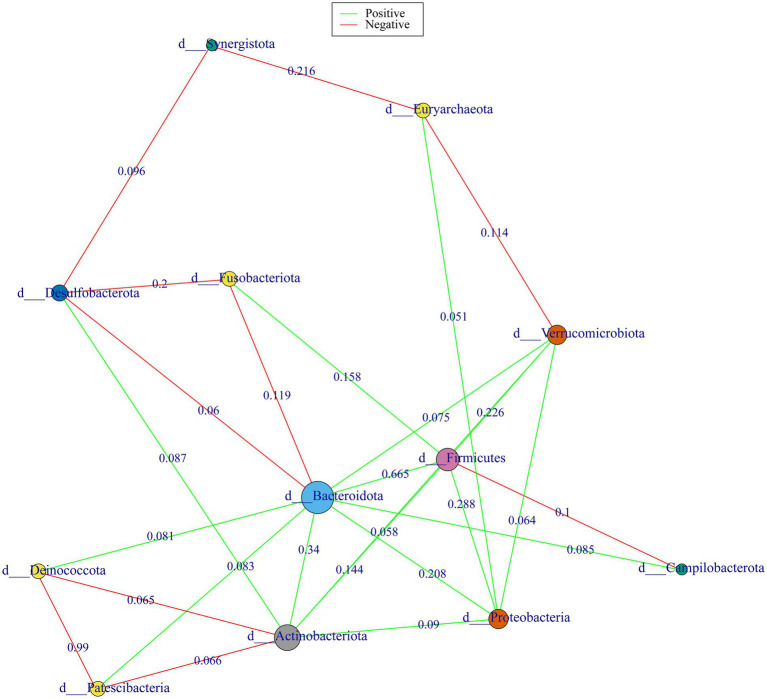
Co-occurrence network analysis of gut microbial phyla in the study. Green edge colors indicate positive correlations and red for negative correlations. Edge weights are labeled with correlation coefficients by Spearman correlation analysis.

The microbial co-occurrence network, constructed from 16S rRNA gene sequencing data, revealed a complex interplay of microbial taxa in phylum level (Figure 5). Node size was proportional to their interaction importance, highlighting key taxa in microbial interactions. *Bacteroidota*, followed by Actinobacteriota showed the most important role in network. Seven microbial taxa have competitive (mutual exclusion) relationships with *Bacteroidota,* including *Actinobacteriota*, *Deinococcota*, *Firmicute*s, *Verrucomicrobiota*, *Proteobacteria*, *Patescibacteria*, and *Campilobacterota*. *Desulfobacterota* and *Synergistota* were cooperative (co-occurence) with *Bacteroidota.*

### 3.6 Differences in microbial metabolic pathways among groups

**Figure 6:**
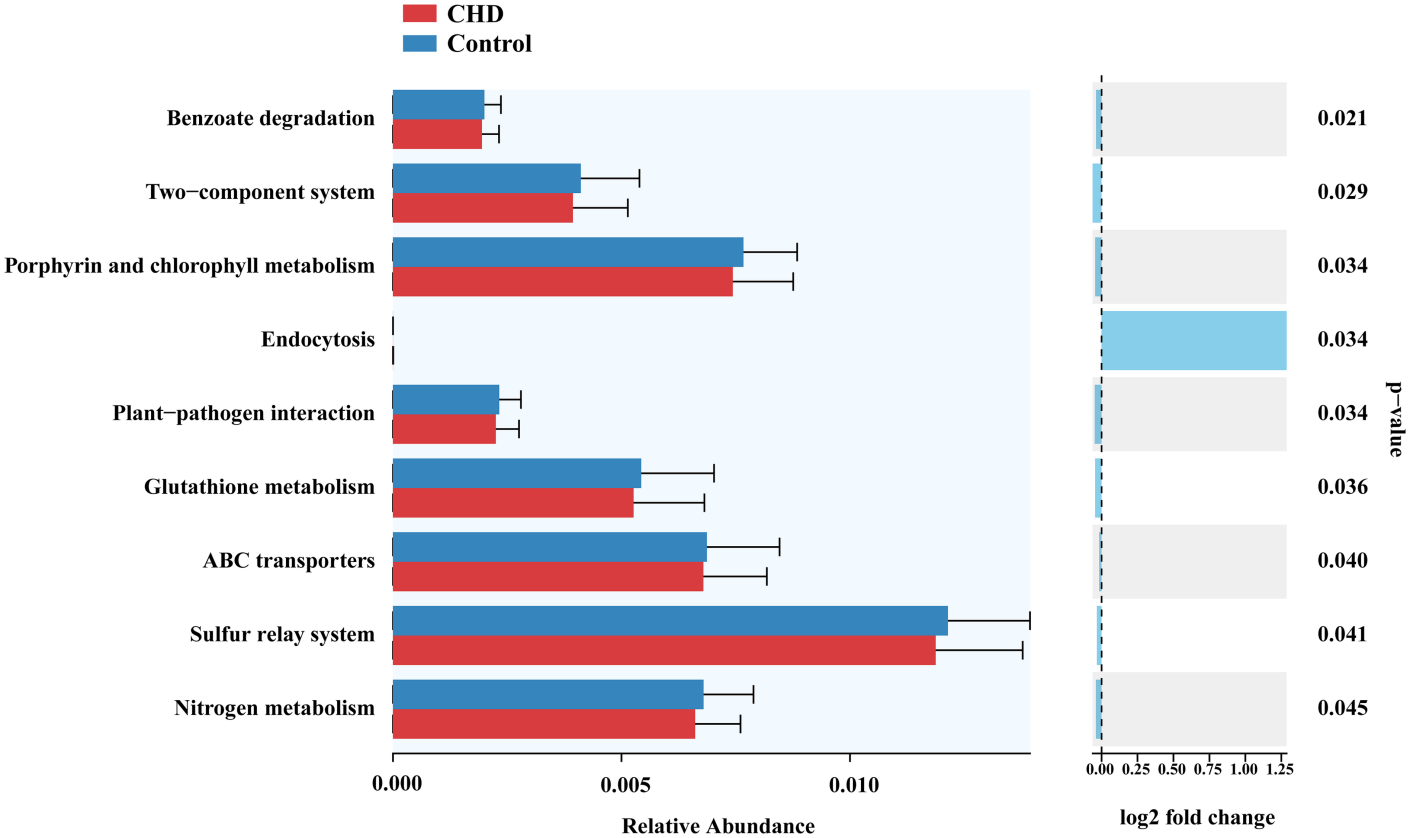
Comparison of KEGG metabolic pathway abundances between coronary heart disease (CHD) patients and controls based on Picrust2 analysis.

To explore functional differences in the gut microbiota between CHD patients and controls, microbial metabolic pathways were predicted using PICRUSt2 based on 16S rRNA gene sequencing data, focusing on KEGG pathways (Figure 6). Compared with controls, the CHD group exhibited significantly lower predicted abundances of several pathways, including, including benzoate degradation (*p* = 0.0216), glutathione metabolism (*p* = 0.0361), porphyrin and chlorophyll metabolism (*p* = 0.0343), nitrogen metabolism (*p* = 0.0454), ABC transporters (*p* = 0.0404), two-component system (*p* = 0.0298), sulfur relay system (*p* = 0.0411), and plant-pathogen interaction (*p* = 0.0348). The predicted downregulation of pathways like glutathione metabolism and porphyrin/chlorophyll metabolism in CHD may reflect altered microbial-host interactions or metabolic dysregulation, warranting further investigation into their roles in coronary pathogenesis. Abundance of Endocytosis was higher (*p* = 0.0344) in CHD group than controls.

### 3.7 Associations among gut microbiota, metabolites and clinical biomarkers

**Figure 7:**
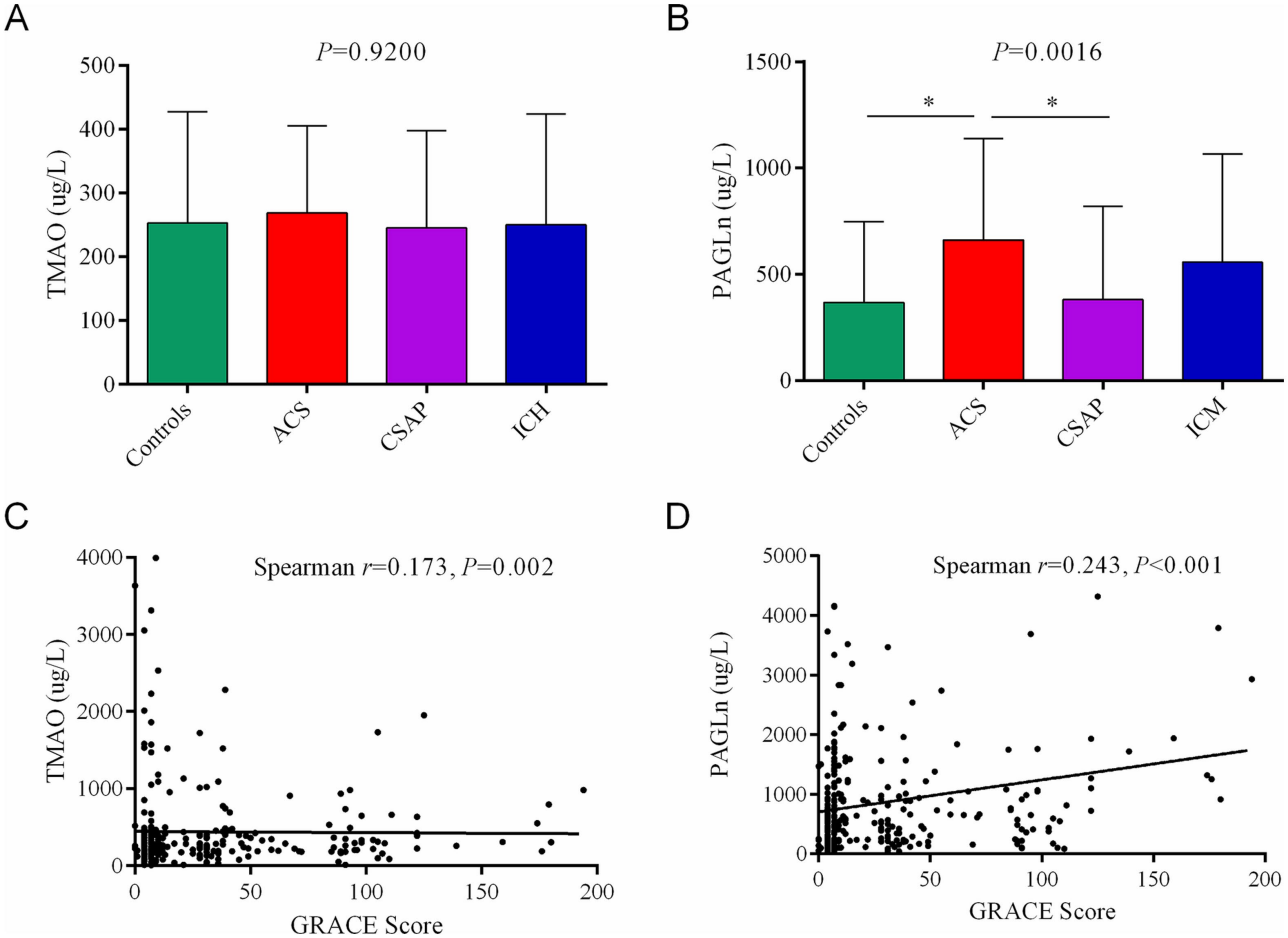
Comparison of gut microbiota-derived metabolites by Kruskal–Wallis *H* test **(A,B)** and their correlations with GRACE scores in coronary heart disease (CHD) subgroups and controls **(C,D)** by Spearman correlation analysis.

Spearman correlation analysis revealed a strong positive association between TMAO and PAGln (Tables 2, *r* = 0.426, *p* < 0.001). TMAO was positively correlated with several clinical markers about glucose metabolism (FBG and HbA1c), Hcy, SCr, ADA and UREA. PAGln showed broader and stronger positive correlations with these markers, such as FBG, HbA1c, Hcy, SCr, LDH, ADA, and UREA (all *p* < 0.001). Notably, PAGln also exhibited negative correlations with several liver function indicators (ALT, GGT, ChE, TP, and ALB). TMAO levels showed no significant differences (Figure 7A, *p* = 0.920), whereas PAGln levels differed significantly (Figure 7B, *p* = 0.0016), with higher levels in the ACS group compared to controls and CSAP. PAGln was more strongly correlated with GRACE score (Figure 7D, Spearman *r* = 0.243, *p* < 0.001) than TMAO (Figure 7C, *r* = 0.173, *p* = 0.002), suggesting a closer association with CHD severity. In the unadjusted linear regression, PAGln was significantly associated with the GRACE score (Table 3, *p* = 0.017). This association remained significant after individual adjustment for sex, diabetes, medication use, or ACS status. However, adjustment for age, BMI or SCr substantially attenuated the association. In the fully adjusted model incorporating all these covariates, the association between PAGln and the GRACE score was no longer statistically significant (*p* = 0.699). Variance inflation factors for all covariates were below 2.0, indicating no substantial multicollinearity. These results suggest that the relationship between PAGln and CHD severity may be confounded by age and renal function in this cohort.

**Table 2:** Spearman correlation analysis between gut microbiota-derived metabolites and biochemical indexes.

Indexes	TMAO	PAGln
TMAO (ug/L)	1.000***	0.426***
PAGln (ug/L)	0.426***	1.000***
Fasting blood-glucose (mmol/L)	0.117*	0.123*
HbA1c (%)	0.151**	0.207***
Hcy (umol/L)	0.123*	0.220***
SCr (umol/L)	0.180***	0.278***
LDH (U/L)	0.035	0.228***
ALT (U/L)	−0.001	−0149**
GGT (U/L)	−0.046	−0.158**
ChE (U/L)	−0.034	−0.150**
TP (g/L)	0.022	−0.116*
ALB (g/L)	−0.043	−0.187***
ADA (U/L)	0.139**	0.233***

*p < 0.05, **p < 0.01, ***p < 0.001.

**Table 3:** Multivariate linear regression analysis of the association between PAGln and GRACE score (*n* = 402) with multicollinearity statistics for the covariates.

Variables	*β* (95% CI)	*p*-value	VIF
PAGln	0.004 (0.001–0.008)	0.017	1.000
PAGln adjusted by sex	0.004 (0.001–0.008)	0.015	1.000
PAGln adjusted by age	0.002 (−0.001–0.006)	0.213	1.088
PAGln adjusted by BMI	0.003 (−0.002–0.008)	0.207	1.010
PAGln adjusted by diabetes	0.004 (0.000–0.007)	0.039	1.029
PAGln adjusted by medications	0.004 (0.001–0.008)	0.021	1.005
PAGln adjusted by ACS	0.004 (0.001–0.008)	0.016	1.004
PAGln adjusted by SCr status	0.003 (0.001–0.007)	0.136	1.381
PAGln adjusted by multiple covariates^a^	0.001 (−0.005–0.007)	0.699	1.612

a Multiple covariates including sex, age, BMI, diabetes, medications, ACS or not, and SCr.

### 3.8 Correlations among gut microbiota, metabolites and immunity

**Figure 8:**
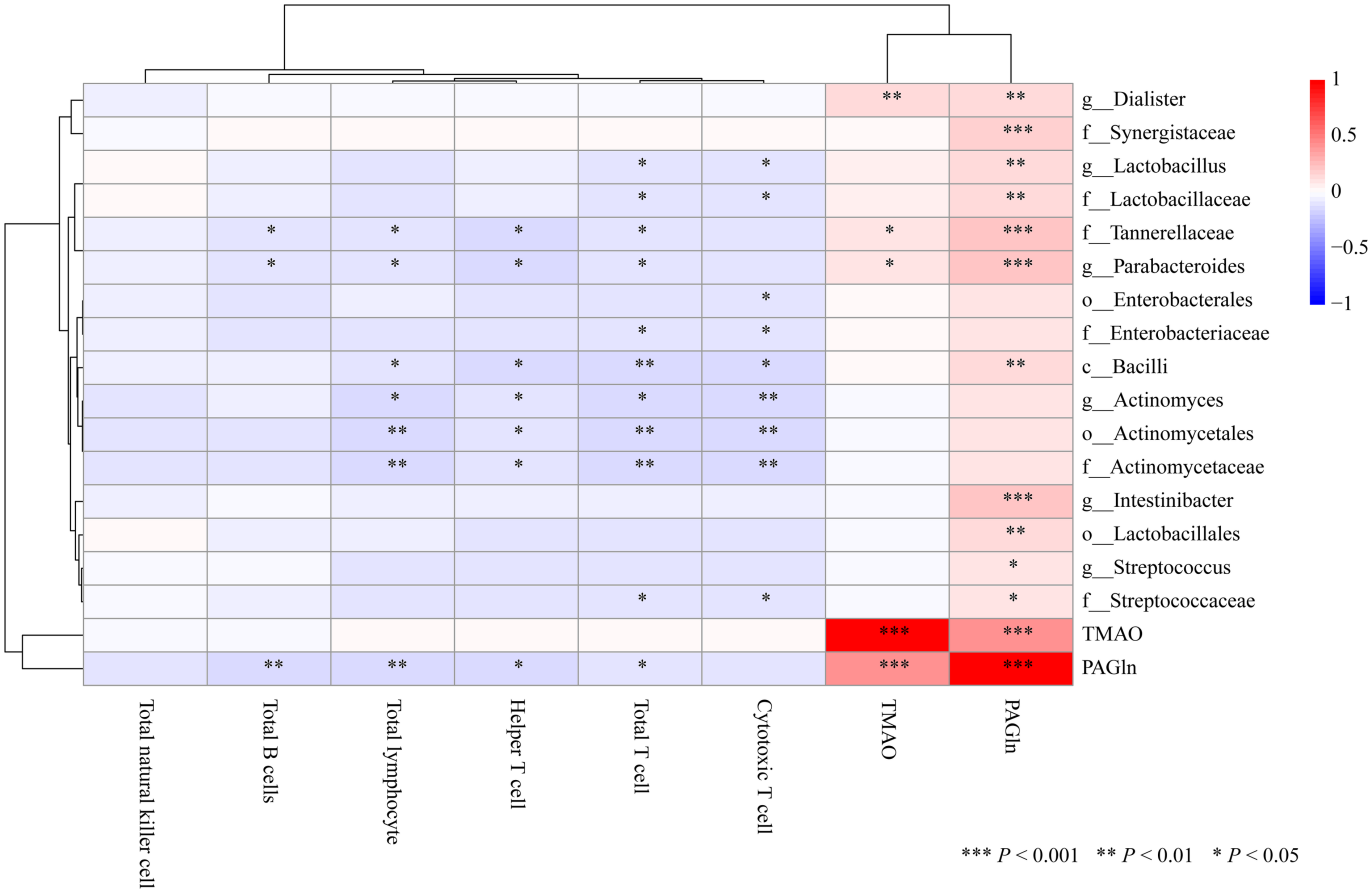
Spearman correlation analysis between gut microbiota taxa, metabolites, and immune cell subsets. Statistical significance is indicated by asterisks: *p < 0.05, **p < 0.01, ***p < 0.001. Positive correlations are shown in red tones, and negative correlations in blue tones.

To explore the interplay between gut microbiota, microbial metabolites, and the host immune system, we conducted Spearman correlation analysis and visualized the results as a heatmap (Figure 8). PAGln showed significant negative correlations with several lymphocyte subsets, including total lymphocytes, total T cells, helper T cells, and total B cells, whereas TMAO exhibited no such associations. These findings suggest that PAGln, rather than TMAO, could have implications for the immune microenvironment in the context of gut microbiota-metabolite interactions. Several gut microbiota taxa inlucidng *Intestinibacter, Parabacteroides, Tannerellaceae, Synergistaceae, Lactobacillus*, and *Dialister*, were strongly associated with PAGln. Most of these taxa were reduced in the CHD group and negatively correlated with immune cell populations such as total lymphocytes, T cells (total, helper, and cytotoxic), and B cells. Together, these findings reveal a complex network linking gut microbiota, metabolites, and immune function. The strong association between PAGln and immune cells, along with taxon-specific microbial changes, suggests that PAGln may act as a key mediator in microbiota-metabolite–immune system crosstalk.

## 4 Discussion

This study provides a comprehensive characterization of gut microbiota dysbiosis, altered microbial metabolites, and associated immune perturbations across distinct subtypes of CHD. By analyzing a sizable cohort of 300 CHD patients-stratified into ACS, CSAP, and ICM—and 100 matched controls, we identified significant alterations in microbial diversity, specific taxonomic profiles, microbial functional capacity, and metabolite levels, especially PAGln. These changes were closely linked to immune cell distributions and clinical severity, suggesting a previously underappreciated gut microbiota-metabolite-immune network in CHD pathogenesis.

In this study, microbial richness, assessed by Chao1 and ACE indices, was significantly reduced across all CHD subtypes compared to controls, with the most marked decrease in ACS patients. However, Shannon and Simpson indices, reflecting overall diversity and evenness, remained unchanged. This partly differ from Jie et al.’s (2017) study, which reported no significant differences in gene richness or diversity between ACVD patients and controls despite changes in dominant and rare genera. LEfSe analysis revealed taxonomic shifts characterized by enrichment of *Actinomycetaceae*, *Streptococcaceae* (genus *Streptococcus*), *Rothia*, and *Dialister*, alongside depletion of beneficial taxa such as *Lactobacillus*. The presence of oral pathobionts like *Rothia* and *Actinomycetaceae* in the gut suggests possible ectopic colonization or systemic translocation driven by oral inflammation-an emerging contributor to atherosclerosis (Xu et al., 2018; Utter et al., 2020). Meanwhile, the reduction in SCFA-producing and immunomodulatory taxa such as *Lactobacillus* implies a weakened gut barrier and a pro-inflammatory microbial profile conducive to endothelial dysfunction (Tang et al., 2019; Wu et al., 2024). PICRUSt2 analysis predicted downregulation of several microbial metabolic pathways in CHD. Of particular interest, glutathione metabolism-a key antioxidant pathway-was suppressed, potentially exacerbating systemic oxidative stress, a possible driver of atherosclerosis (Hristov, 2022). Nitrogen metabolism, another downregulated pathway in our study, has also been implicated in fatty liver disease (Delgado et al., 2022). Disruption of this pathway and impaired gut-liver metabolic communication may lead to hepatic ammonia accumulation, contributing to steatosis and potentially progressing to hyperammonemia. It should be noted that these functional alterations were inferred from 16S rRNA gene-based predictions. Validation using shotgun metagenomic sequencing or metabolomic analyses will be necessary to confirm whether these predicted pathway differences translate into actual metabolic alterations in CHD.

A pivotal and novel finding is the marked elevation of plasma PAGln in CHD patients-especially those with ACS. PAGln levels positively correlated with the GRACE score in unadjusted analysis, a validated index of ACS severity, underscoring its potential utility as a more sensitive biomarker than TMAO for stratifying CHD risk. This is in line with the findings of Nemet et al., (2020), who reported PAGln as a gut-derived, phenylalanine-based metabolite with pro-thrombotic effects via adrenergic receptor signaling (Nemet et al., 2020). Haoran Wei et al. demonstrated that increased plasma PAGln independently forecasts a greater likelihood of adverse cardiovascular events among heart failure patients (Wei et al., 2023). However, after adjusting for key clinical confounders, the association between PAGln and GRACE score was no longer statistically significant, suggesting that the observed correlation may be substantially influenced or confounded by age and renal function. Notably, PAGln has been reported to be cleared from the body via renal excretory function (Poesen et al., 2016), which is linked to cardiovascular risk and disease severity (Jankowski et al., 2021). Therefore, the observed association between PAGln and CHD severity likely reflects its integration within a broader cardiometabolic and cardiorenal context rather than a fully independent effect. These findings underscore that PAGln may serve as a biomarker reflecting systemic metabolic dysfunction, rather than a standalone predictor of CHD severity. Most strikingly, elevated PAGln levels were significantly negatively associated with lymphocyte subsets, including total lymphocytes, T cells, and B cells. This finding extends PAGln’s potential role beyond thrombosis, suggesting an association with altered lymphocyte counts. Our correlation network reveals that enrichment of specific taxa (e.g., *Parabacteroides*, *Tannerellaceae*) was associated with both elevated PAGln and reduced lymphocyte counts. This may support a tripartite model linking microbial dysbiosis, increased PAGln, and lymphocyte counts reductions. Additionally, modulating the gut microbiota to reduce PAGln production represents a novel therapeutic strategy. Targeted interventions-such as fiber-rich diets, prebiotics, probiotics, or even fecal microbiota transplantation—may help restore a favorable microbial balance. Moreover, modulation of PAGln production may represent a potential area for future research.

Notably, in contrast to prior studies linking TMAO to atherosclerotic cardiovascular disease, we did not observe significant differences in TMAO levels among CHD subgroups and controls. Seminal studies by Stanley L. Hazen demonstrated that elevated TMAO promotes platelet hyperreactivity and atherosclerosis, and multiple epidemiological studies have associated higher TMAO levels with adverse cardiovascular outcomes (Zhu et al., 2016; Wang et al., 2011; Tang et al., 2013). Several factors may account for the discrepancy between our findings and previous reports. First, TMAO levels are influenced by dietary choline and carnitine intake (Thomas and Fernandez, 2021; Koeth et al., 2013), which were not assessed in the present study. In addition, some patients in our cohort were receiving cardiovascular medications, and previous reports have suggested that statins may reduce circulating TMAO levels (Jamialahmadi et al., 2025). Besides, TMAO is primarily cleared by the kidneys, circulating TMAO levels are dependent on renal function (Missailidis et al., 2016). We also found TMAO level was positively correlated with SCr level, which reflects renal function to a certain extent. Variability in renal clearance among participants may have contributed to the absence of significant group differences observed in the present study. Importantly, several prior clinical studies have reported similar observations, supporting the findings of the present study. Hao Liang et al. reported that TMAO levels did not significantly differ between patients with atherosclerosis and healthy controls, whereas diabetes exerted a significant impact on plasma TMAO levels (Liang et al., 2023), which is consistent with our findings. Taken together, these results suggest that the contribution of TMAO to CHD may be substantially influenced by diet, renal function, medication exposure, and population characteristics, underscoring the importance of considering these confounders when interpreting TMAO–cardiovascular associations.

Several limitations should be noted. First, the cross-sectional design precludes causal inference. Longitudinal studies are needed to establish whether gut dysbiosis and PAGln elevation precede CHD onset. Second, functional inference via PICRUSt2 lacks the resolution of metagenomic sequencing. Third, our immune profiling was limited to broad lymphocyte categories, warranting more detailed phenotyping (e.g., Tregs, Th17) and functional assays. Therefore, we cannot distinguish between true functional immunosuppression and numerical lymphopenia. In addition, dietary data—which critically influence gut microbiota—was not collected. Besides, due to complex multidrug regimens of CHD patients, this study cannot fully analyze drug-specific effects on gut microbiota, metabolites, or immune parameters. This single-center study requires validation in multi-center, multi-ethnic cohorts.

## 5 Conclusion

This study reveals a multi-dimensional disruption in gut microbiota composition and predicted function across CHD subtypes, characterized by reduced microbial richness, pathobiont enrichment, functional shifts, and elevated PAGln levels. Although PAGln was not independently associated with GRACE score after full adjustment, it was linked to immune alterations, gut microbial dysbiosis, and disease severity. These findings highlight coordinated changes among gut microbiota, microbial metabolites, and host immunity in CHD and support further investigation into the role of microbial metabolism in cardiovascular disease.

## Data Availability

The data presented in this study are publicly available. The data can be found at: https://www.ncbi.nlm.nih.gov, accession PRJNA1277534.
